# Coprophagy Prevention Affects the Reproductive Performance in New Zealand White Rabbits Is Mediated through Nox4-ROS-NF*κ*B Pathway

**DOI:** 10.1155/2022/8999899

**Published:** 2022-12-21

**Authors:** Zhichao Li, RuiTing Li, Jing Li, Zhitong Wang, Hui He, Duo Yan, Lei Yu, Hengjian Li, Ming Li, Huifen Xu

**Affiliations:** ^1^College of Animal Science and Technology, Henan Agricultural University, Zhengzhou 450046, China; ^2^Animal Health Supervision Institute of Biyang, Henan 463700, China

## Abstract

Coprophagy is of great significance to the growth, development, and reproductive performance of rabbits. This study is aimed at exploring the effect of coprophagy on the reproductive performance of New Zealand white rabbits by coprophagy prevention (CP). The results showed that CP treatment significantly decreased the growth and development performance of female rabbits and the live birth rate of embryos. The results of blood biochemical indexes showed that CP treatment significantly increased the contents of serum ALB, ALP, and MDA, while serum SOD activity was significantly decreased. Transcriptome analysis showed that GO terms were mainly enriched in transport function and reproductive function after CP treatment. In addition, KEGG results showed that inflammation related signal pathways were activated and the expression level of genes related to tight junction proteins was downregulated by CP treatment. Concurrently, western blot further confirmed the results of KEGG. In short, fecal feeding is an important survival strategy for some small rodents, coprophagy prevention will affect the inflammatory level of the body, change the oxidative stress level of the body, and then activate NOX4-ROS-NF-*κ*B pathway, increase the expression level of adhesion protein ICAM-1 and VCAM-1, lead to the damage of uterine epithelial barrier, and then affect the reproductive performance of rabbits.

## 1. Introduction

Coprophagy refers to the behavior of animals feeding on feces, which is very common in small and medium nonhuman primates [1, 2]. Coprophagy behavior is a nutritional adaptation strategy for small and medium-sized herbivores to low quality food. On the one hand, coprophagy is helpful to the recovery and establishment of animal intestinal microbiota, on the other hand, it can provide the host with the energy and nutrition needed for growth and development. [2–5]. Intestinal microbiota is mutually beneficial to the host and plays an important role in the growth and development, reproduction, immune regulation, and other physiological functions of the host [6–8]. Intestinal microbiota, mucus barrier, epithelial barrier, and immune barrier constitute intestinal barrier to maintain intestinal homeostasis [9]. Destruction of intestinal homeostasis can cause inflammatory bowel disease (IBD), irritable bowel syndrome (IBS), colorectal inflammation, cancer, and other diseases [10–13]. Therefore, intestinal microbiota has been considered as a necessary “immune organ” for human and animals to survive and maintain health [14]. In addition, the gut can interact with a variety of organs through a complex neural immune endocrine network, forming a relationship similar to the “axis”, that is, the gut organ axis [15].

The liver is the first recipient of intestinal metabolites, receiving small dietary nutrient metabolites (such as amino acids and short chain fatty acids) and other small molecular metabolites absorbed by the intestinal mucosa [16–18]. At the same time, the liver can secrete bile acids into the intestine. Most of these bile acids are reabsorbed from the ileum, transported back to the liver through the portal vein, and then secreted back to the bile duct. This process is called enterohepatic cycle [19, 20]. These two-way connections between the intestine and the liver are the bridge between the intestine microbiota and the liver axis, and they are the important structural basis for the intestine liver axis to regulate the nutritional metabolism and immunity of animals [21–24]. The hepato intestinal circulation of bile acids is a key factor in the interaction between intestine and liver [25, 26]. Bile acid regulates the metabolic pathway and inflammatory response of the body through Farnesoid X receptor (FXR) and G protein coupled bile acid receptor 1 (Gpbar1 or TGR5) [27]. Intestinal microbiota interacts with bile acids in both directions [26]. Bile acids affect the composition of intestinal microbiota by shaping intestinal immunity and some endogenous antibacterial properties [28]. Intestinal microbiota can metabolize bile acid into secondary bile acids, so the change of intestinal microbiota composition will break the balance between primary bile acid and secondary bile acids in the body, thus promoting the progress of liver disease [29, 30]. Moreover, the intestinal barrier can effectively prevent intestinal microorganisms and the harmful substances generated by their metabolism from entering the liver. When the intestinal barrier is damaged, the harmful substances in the intestine will invade the liver and participate in the occurrence and development of liver and related organ diseases [18, 31–34]. While the function of liver also has an important impact on maternal reproductive performance. The ovulation process of female birds will be accompanied by the production of a large number of reactive oxygen species ROS. With the increase of age, SOD activity will gradually decrease, which will lead to the continuous accumulation of ROS, further cause follicular atresia, and directly lead to the decline of ovarian function [35–37]. Therefore, oxidative stress is an important reason for the decline of female reproductive performance [38, 39].

The previous research results of our research group showed that CP caused changes in microbiota in the cecal contents of rabbits, reduced lipid metabolism in the liver, and affected growth performance [40–42]. Song et al. demonstrated the detrimental effect on the reproduction of the rabbit by preventing coprophagy with a main role for this response played by CTSB on the granulosa cells of the ovary [43]. In addition, studies have reported that CP can cause fetal dysplasia in pregnant mice [44]. The frequency and intake of fecal feeding in mice during pregnancy and lactation increased significantly, indicating that coprophagy behavior has nutritional significance during pregnancy [45]. However, the mechanism of the effect of fecal feeding behavior on host reproductive performance is still unclear. When sequencing at the transcriptome level, a species will have significant differences in a specific trait under different treatment conditions. With individuals with significant differences as controls, it can often be used as a good material to find and study the regulatory factors related to specific traits [46, 47]. Therefore, this study used high-throughput sequencing technology to sequence the transcriptome of endometrial epithelial cells of New Zealand white rabbits who were CP or not, aiming to reveal the regulatory mechanism of coprophagy behavior on the reproductive performance of New Zealand white rabbits.

## 2. Materials and Methods

### 2.1. Experimental Design, Animals and Management

**Figure 1:**
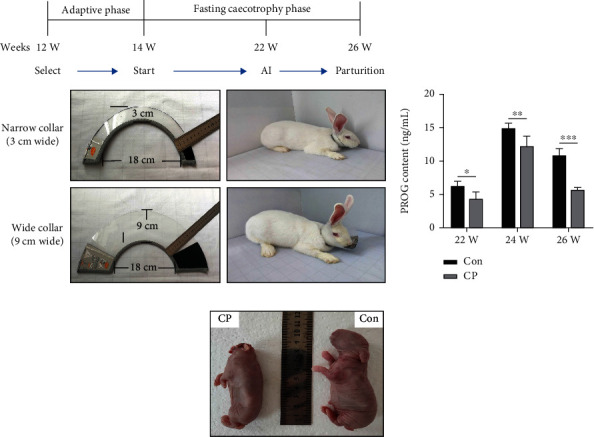
Experimental design. (a) Timeline of studies and fasting soft feces tools. (b) PROG (Progesterone) content. (c) Newborn rabbits in Con and CP group.

Sixty 14-week-old female New Zealand white rabbits of similar weight were provided by Jiyuan sunshine rabbit Technology Co., Ltd. All New Zealand white rabbits were randomly divided into two groups, with 30 New Zealand white rabbits in each group. Among them, New Zealand white rabbits in CP group wore a collar with a width of 9 cm to prohibit them from eating feces (Figure 1(a)), and New Zealand white rabbits in Con group wore a collar with a width of 3 cm to allow them to eat feces (Figure 1(a)). Twelve individuals (CP1-6 and Con1-6) were randomly selected from 60 New Zealand white rabbits. Both the CP group and the Con group contained six independent biological replicates, avoiding the impact of extreme individual differences on the experimental data. These New Zealand white rabbits are kept in cages alone and have free access to feed and water. Using the same rabbit breeds (rabbits of the same age and sex (female)), basic dietary ingredients, feeding methods, and environment, a sampling rate of 20% can ensure the high reliability of the data. All rabbits are raised according to the appropriate guidelines for raising rabbits. The diet composition of rabbits was shown in Supplementary Table S1.

### 2.2. Determination of Growth and Development, Reproductive Performance and Serum Indexes

During the experiment, the growth and development indexes of two groups of New Zealand white rabbits were measured, including initial weight, average daily intake, average daily gain, premortem live weight, and feed conversion ratio. After the experimental animals were cultured for 22 weeks, artificial insemination and breeding were carried out, and the reproductive performance of the two groups of New Zealand white rabbits was measured after delivery (after 26 weeks) (Experiment design is shown in Figure 1(a)). Then tissue sampling was carried out. First, pentobarbital sodium was used for anesthesia, and intravenous bleeding was performed for euthanasia. Part of the blood samples were collected to determine the serum biochemical indicators of the two groups of New Zealand white rabbits. The slaughtering performance of two groups of New Zealand white rabbits was determined.

### 2.3. Sample Collection of Uterine Tissue and Endometrial Epithelial Cells

Collect fresh uterine tissue and endometrial epithelial cells, wash them with newly prepared DEPC water, collect the above samples in sterile EP tubes without RNAse-/DNAse, and store them at -80°C for RNA-seq (In RNA-seq, we randomly selected 3 samples from the Con and CP groups, respectively).

### 2.4. Extraction and Quality Assessment of Total RNA from Endometrial Epithelial Cells and Library Construction

Total RNA was extracted from endometrial epithelial cells of New Zealand white rabbits by Trizol method, and then the concentration and quality of the extracted RNA were measured by NanoDrop spectrophotometer (Thermo Scientific), and the integrity of RNA was analyzed by Agilent 2100 biological analyzer. A sequencing library was generated using truseq RNA sample preparation kit (Illumina, San Diego, CA, USA). After passing the quality inspection, the library was sequenced on the HiSeq platform (Illumina) by Bioyigene Biotechnology Co., Ltd. (Wuhan, China).

### 2.5. Sorting, Filtering, and Bioinformatics Analysis of Transcriptome Sequencing Data

After sequencing, the raw data of each sample is counted separately. In addition, the sequencing data often contains some low-quality reads with connectors. These sequences will cause great interference to the subsequent information analysis, so it is necessary to further filter the sequencing data. The standards of data filtering mainly include: (1) use cutadapt to remove the connector at the 3′ end, and the removed part has at least 10 bp overlap (AGATCGGAAG) with the known connector, allowing 20% base mismatch. (2) Remove the reads whose average mass fraction is lower than Q20. Then, HISAT2 software [48] was used to compare the filtered data with the reference genome (OryCun2.0). According to the comparison results, HTseq (0.11.3) software [49] was used to calculate the expression of each gene. On this basis, the samples were analyzed by expression quantity analysis, expression difference analysis, and cluster analysis, and the relevant pictures were drawn by the R Programming Language (3.6.1) and Python (2.1) and other analysis software.

### 2.6. Levels of Oxidative Stress in Endometrial Epithelial Cells

Firstly, the expression levels of genes related to mitochondrial oxidative stress in the transcriptome were analyzed, the mitogenomic map was depicted with CG View Server (http://stothard.afns.ualberta.ca/cgview_server/) [50]. And then the contents of NADPHt, NADPH, and NADP+ in endometrial epithelial cells were detected by NAD^+^/NADH detection kit (WST-8 method, Beyotime, #S0175), and the levels of ROS in endometrial epithelial cells were detected by reactive oxygen species detection BBoxiProbe® DHE kit (Bestbio, #BB-470515).

### 2.7. Analysis of Inflammatory Level and Barrier Function of Endometrial Epithelial Cells

For total protein extraction, RIPA lysis buffer (PC101, Epizyme, China) with protease inhibitor cocktail (GRF101, Epizyme, China) and phosphatase inhibitors mixture (GRF101, Epizyme, China) was utilized to lyse the tissues. Then, the lysate was centrifuged at 12,000 g, 4°C for 20 min to obtain the total protein. The protein concentrations were detected by the BCA Protein Assay Kit (ZJ101, Epizyme, China) and western blot was performed using sodium dodecyl sulfate polyacrylamide gel electrophoresis (SDS-PAGE) method. The transferred membranes were incubated at 4°C overnight with the following primary antibodies: IL-17 (1 : 500, #13082-1, Proteintech), TNF-a (1 : 500, #60291-1, Proteintech), NOX4 (1 : 1000, GB11347, Servicebio), p-IkBa (1 : 500, BS4105, Bioworld), IkBa (1 : 500, MB0106, Bioworld), NF*κ*B (1 : 1000, GB12142, Servicebio), ICAM1 (1 : 1000, #4915, CST), and GAPDH (1 : 1000, #2118, CST). The membranes were then incubated with the corresponding secondary antibodies, including HRP linked anti-mouse IgG (1 : 2000, #7076, CST), HRP linked anti-rabbit IgG (1 : 2000, #7074, CST) for 2 h at room temperature. The blots were visualized using LAS4000 chemiluminescence system (Fujifilm, Tokyo, Japan), and the densities were analyzed by ImageJ 1.8 software.

### 2.8. Verification of RNA-Seq Results by RT-qPCR

In this study, RT-qPCR was used to verify the accuracy of transcriptome sequencing data. First, six genes were randomly selected from the weight of differentially expressed genes, including three upregulated genes, two downregulated genes, and one gene with no difference in expression. The RNA of the above five genes was used as a template for RT-qPCR analysis, and the primers of these six genes were designed on NCBI. See Supplementary Table S2 for primers information. Use the reverse transcription kit to reverse transcribe the above RNA into cDNA on the RT-PCR instrument, and then conduct qPCR. Supplementary Table S3 was for the amplification system and amplification conditions of qPCR, and all reactions are in triplicate. The gene expression data were standardized by 2-∆∆CT method with reference genes, and were statistically analyzed.

### 2.9. Statistical Analysis

A completely randomized trial design was used. The data were analyzed by *Studentst*-test of independent samples in SPSS 24.0. ^∗^ means significant difference, that is, *P* value < 0.05, ^∗∗^ means extremely significant difference, *P* value < 0.01, NS means no significant difference between the data, that is, *P* value > 0.05.

## 3. Results

### 3.1. Results of Growth and Development, Reproductive Performance and Serum Indexes

**Figure 2:**
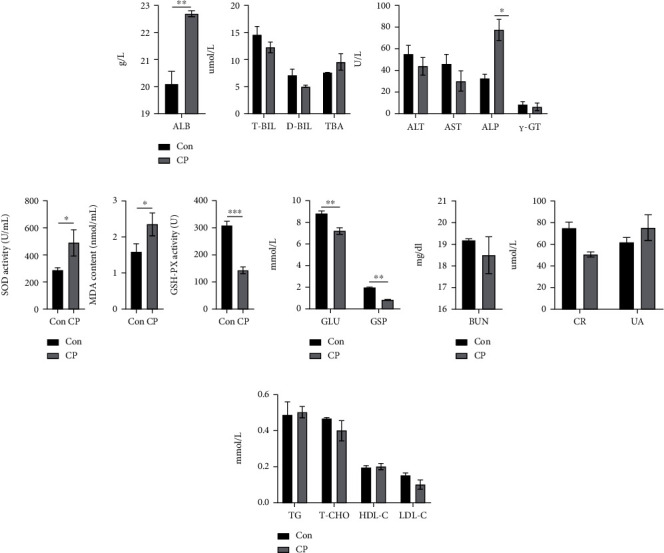
Comparison of serum biochemical parameters between Con and CP groups. (a) Contents of albumin, total bilirubin, direct bilirubin, total bile acid alanine aminotransferase, aspartate aminotransferase, alkaline phosphatase, and glutamyl transpeptidase. (b) Superoxide dismutase and glutathione peroxidase activities, malondialdehyde content. (c) Content of glucose and glycosylated serum protein. (d) Content of blood urea nitrogen, blood creatinine, and blood uric acid. (e) Contents of triglycerides, total cholesterol, high-density lipoprotein cholesterol, and low-density lipoprotein cholesterol.

The growth performance indicators of the two groups of New Zealand white rabbits are shown in Table 1. After CP, the average daily feed intake, average daily body weight gain, and final weight of the CP group are significantly lower than those of the Con group, but the ratio of feed intake/body weight is elevated in the CP group. (*P* value < 0.05). There is no significant difference between the two groups in initial weight and average daily intake (*P* value > 0.05). The effects of coprophagy prevention on rabbit slaughter performance is shown in Table 2. The all-eviscerated weight, half eviscerated weight, and uterus weight of rabbits in the CP group are significantly lower than those in the control group (*P* value < 0.05), while the oophoron weight and placenta weight of New Zealand white rabbits in the two groups have no significant difference (*P* value > 0.05) (Table 2). The effect of CP on the reproductive performance of New Zealand white rabbits is shown in Table 3. The breeding rate, live birth rate, stillbirth rate, and birth litter weight of rabbits in the CP group are significantly lower than those in the control group (*P* value < 0.05), while the total number of births, live births, and birth weight of New Zealand white rabbits in the two groups have no significant difference (*P* value > 0.05) (Table 3). The influence results of serum biochemical indexes of New Zealand white rabbits in the CP group after CP are shown in Figure 2. The contents of ALB, ALP, and MDA of New Zealand white rabbits in the CP group are significantly higher than those of New Zealand white rabbits in the control group, while the contents of SOD activity, GSH-Px activity, Glu, and GSP are significantly lower than those of New Zealand white rabbits in the control group.

### 3.2. Quality Control and Expression Analysis of Transcriptional Sequencing Data

Transcriptome results showed that the comparison rate of filtered reads of each sample was greater than 89%, and the Q30 value was greater than 92% (Supplementary Table S4). HTSeq was used to calculate the original expression of genes and FPKM was used to standardize the expression. The expression pattern of all genes in the sample were performed by violin diagram. The distribution of gene expression of the six samples was shown in Supplementary Figure S1A. The distribution of gene expression ranged from -2.5 to 2.5, and the median of gene expression was about 1. Pearson correlation coefficient is used to express the correlation of gene expression levels between samples. The closer the correlation coefficient is to 1, the higher the similarity of expression patterns between samples. The results showed that the higher the similarity of intragroup expression patterns of the two groups of samples, the lower the similarity of intergroup expression patterns (Supplementary Figure S1B). What is more, using the DESeq software package of R language, PCA principal component analysis was carried out on each sample according to the expression volume. The PCA results showed that the three samples in the control group were closer, while the three samples in the CP group were farther away, and the distance between the two groups was also farther. The distance showed the similarity between samples (Supplementary Figure S1C). The above results showed that this transcriptome sequencing has high accuracy and reliable quality, which meets the requirements of transcriptome analysis.

### 3.3. Verification of Transcriptome Sequencing Results by RT-qPCR

The relative expression results of FPKM and RT-qPCR of transcriptome data are shown in the Supplementary Figure S2. The expression of the six genes identified by RT-qPCR was consistent with the FPKM of the transcriptome data, and the folding changes were basically the same. RT-qPCR was consistent with the transcriptome data, indicating the accuracy of the transcriptome sequencing results.

### 3.4. Differentially Expressed Gene Analysis

**Figure 3:**
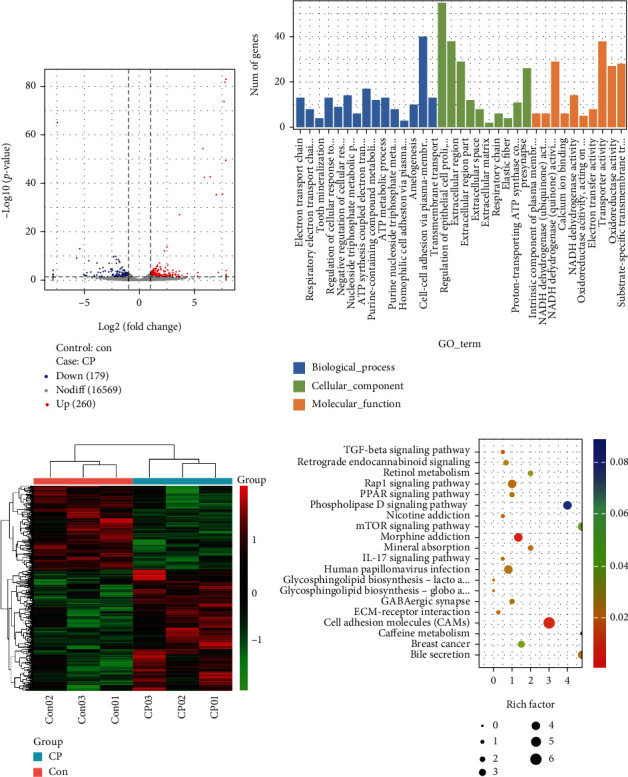
Analysis of differentially expressed genes between Con and CP groups. (a) Display differentially expressed genes in the form of volcano map, including upregulated genes (red), downregulated genes (blue), and no differentially expressed genes (gray). (b) The clustering pattern analysis of differentially expressed genes. (c) GO terms enrichment analysis of differentially expressed genes. (d) KEGG pathway enrichment analysis of differentially expressed genes.

By comparing the expression levels of all genes in the CP group and the Con group, a total of 439 differentially expressed genes (DEGs) were screened, including 260 upregulated genes and 179 downregulated genes (Figure 3(a), Supplementary Table S5).  Figure 3(b) shows the clustering pattern analysis of DEGs between the CP and the Con group. The results showed that the DEGs of each samples selected from the CP group or the control group show a similar expression pattern.

### 3.5. GO Function Enrichment Results for Differentially Expressed Genes

GO notes divided 439 DEGs into three categories: biological processes (BP), cellular components (CC), and molecular functions (MF). The first three GO terms with the most significant enrichment obtained from the DEG annotation are extracellular domain (GO:0005576), electron transport chain (GO:0022900), and NADH dehydrogenase (ubiquinone) activity (GO:0008137) (Figure 3(c)). Further analysis showed that GO terms related to transport function and reproductive function were significantly enriched in 640 GO terms (*P* value < 0.05 or *P* value < 0.01) (Supplementary Table S6).

### 3.6. KEGG Pathway Enrichment Results for DEGs

The KEGG classification annotation results of DEGs are shown in Supplementary Table S7. 439 DEGs are labeled in the five primary levels of KEGG: metabolism, environmental information processing, tissue systems, cellular processes, and human diseases. As shown in the scatter diagram in Figure 3(d), further KEGG enrichment analysis identified the top 20 KEGG pathways. According to the chart, the main enriched KEGG pathways are morphine addiction, cell adhesion molecules, ECM receptor interaction, Rap1 signaling pathway, melanogenesis, IL-17 signaling pathway, and NF-*κ*B signal pathway.

### 3.7. Oxidative Stress State of Endometrial Epithelial Cells

**Figure 4:**
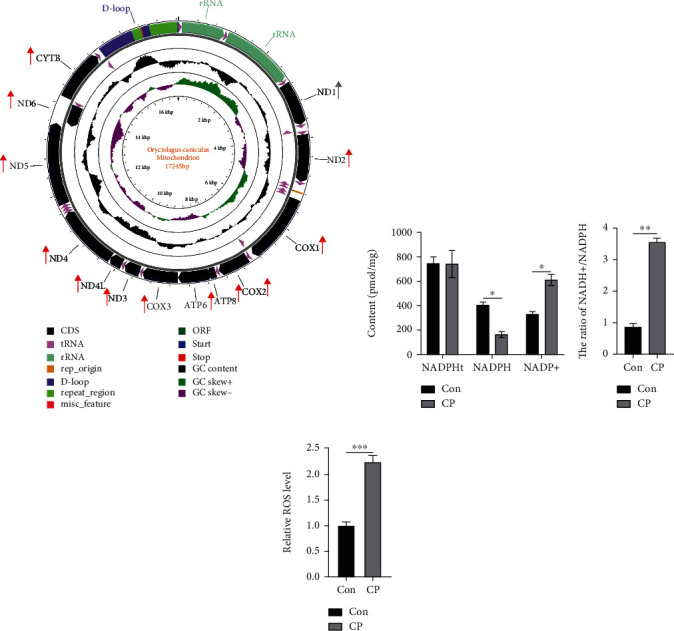
Oxidative stress state of endometrial epithelial cells between Con and CP groups. (a) The map of mitochondria was drawn with CGView software, and then the genes of mitochondria were marked according to the results of transcriptome sequencing. These genes were all upregulated. The red ones represented significant (*P* value < 0.05), and the gray ones represented no significant (*P* value > 0.05). B&C NADPHt, NADPH, and NADP+ content and NADH+/NADPH value. (d) Reactive ROS level between Con and CP group.

The map of mitochondria was drawn with CGView software, and then the genes of mitochondria were marked according to the results of transcriptome sequencing. These genes were all upregulated, with red representing significant (*P* value < 0.05) and gray representing no significant (*P* value>0.05). The results showed that after CP, the expression of epoxidases COX1, COX2, and COX3 were upregulated, and the expression of NADH dehydrogenase family such as ND1-6 gene was upregulated (Figure 4(a)). In addition, CP treatment significantly increased the NADP+ content, NADP+/NADPH ratio and reactive oxygen species (ROS) level in endometrial epithelial cells (Figures 4(b), 4(c), and 4(d)).

### 3.8. Inflammation Level and Barrier Function Results

**Figure 5:**
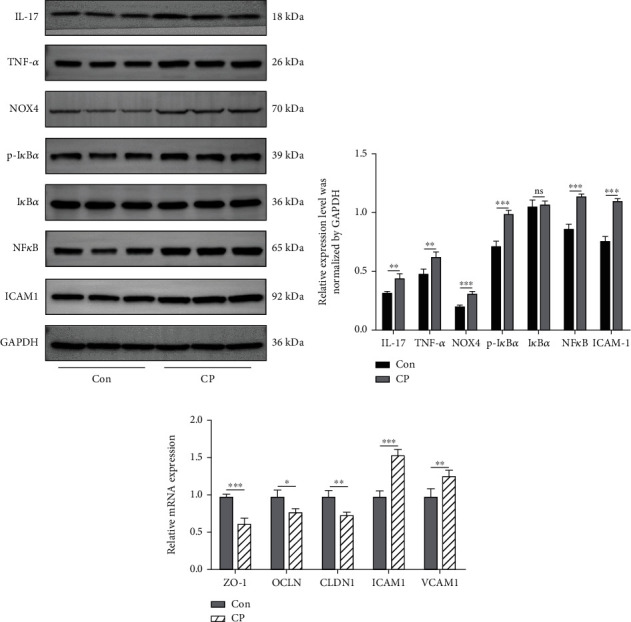
Inflammation level and barrier function verification of endometrial epithelial cells. (a) Western blot results of inflammatory and adherence factors in endometrial epithelial cells of Con and CP groups. (b) Relative expression levels were normalized by GAPDH (Inflammatory and adherence factors). (c) RT-qPCR results of barrier function and adherence factors in endometrial epithelial cells of Con and CP groups.

Western blot results showed that the expression levels of TNF-*α*, IL-17, and NF-*κ*B signal pathways related to inflammation in endometrial epithelial cells were significantly increased (*P* value < 0.05 or *P* value < 0.01) (Figures 5(a) and 5(b)). RT-qPCR results demonstrated that the relative expression levels of *ZO-1*, *Occlaudin* and *Claudin-1* related to endometrial epithelial barrier were significantly decreased (*P* value < 0.05 or *P* value < 0.01) (Figure 5(c)). What is more, the expression levels of vascular cell adhesion molecule-1 (*VCAM-1*) and intercellular adhesion molecule-1 (ICAM-1) in endometrial epithelial cells of CP group were significantly increased (Figures 5(a), 5(b) and 5(c)).

## 4. Discussion

The previous research results of our research group showed that CP caused changes in microorganisms in the cecal contents of rabbits, reduced lipid metabolism in the liver, and affected growth performance [40]. The results of the present study showed that CP significantly reduced the breeding rate, live birth rate, and birth litter weight of New Zealand white rabbits. In addition, CP significantly reduced the content of progesterone (7th d and 21st d) in New Zealand white rabbits. Progesterone is secreted by the corpus luteum of the ovary, which has a significant morphological effect on the endometrium stimulated by estrogen in the body and is necessary to maintain pregnancy [51, 52]. Studies have shown that increasing the content of progesterone in rabbits can significantly increase the pregnancy rate of rabbits [53, 54]. In this study, CP significantly reduced the progesterone content of New Zealand white rabbits, which may be the reason for the low breeding rate. On the other hand, the effect of CP on the serum parameters of New Zealand white rabbits significantly increased the contents of ALB, ALP, and MDA in the serum. The abnormal increase of serum ALB and MDA suggested that inflammatory factors were activated, and the abnormal increase of ALP may be related to the decrease of nutrients in blood, while the increase of SOD activity may lead to the continuous accumulation of ROS and oxidative stress (Figure 4). In addition, there have been relevant reports on poultry, so it is speculated that the decline in reproductive performance of CP group is due to inflammation and endoplasmic reticulum pressure [55].

Among the differentially expressed genes in the transcriptome, fasting soft stool significantly reduced the expression level of FGF18 and LIF genes. Fibroblast Growth Factor 18 (FGF18) is a kind of polypeptide molecule that plays a role by binding to specific receptors of cell membrane [56]. In the embryonic study of FGF18 knockout mice, all FGF18 knockout mice died in the neonatal period, and the bone development was abnormal [57]. Leukemia inhibitory factor (LIF) is a pleiotropic glycoprotein cytokine of interleukin-6 (IL-6) cytokine family. Leukemia inhibitory factor (LIF) is a multifunctional cytokine, which plays an important role in early embryonic development and implantation. LIF plays its biological role through leukemia inhibitory factor receptor (LIFR). Targeted destruction of LIFR gene leads to placental, skeletal, neural and metabolic defects and perinatal death [58]. The above results indicate that fasting soft feces may affect the reproductive performance of rabbits by destroying the expression level of FGF18 and LIF genes.

GO function enrichment results show that GO terms related to transport function, such as transmembrane transport (GO: 0055085); transporter activity (GO: 0005215), substrate-specific transmembrane transporter activity (GO: 0022891), organic anion transmembrane transporter activity (GO: 0008514), carboxylic acid transmembrane transporter activity (GO: 0046943), transmembrane transporter activity (GO: 0022857) and fatty acid transport (GO: 0015908) are significantly enriched. Research showed that animals can obtain more nutrients by coprophagy, which prolongs the time of feed passing through the digestive tract [1, 59]. Moreover, it improves the digestion and absorption rate of feed and plays an important role in promoting the growth and development of embryos [60]. However, after CP, for pregnancy rabbits, the transport capacity of uterus to nutrients is down-regulated, affecting the growth and development of embryos. In addition, GO terms related to reproductive function, such as reproductive structure development (GO: 0048608), reproductive system development (GO: 0061458), developmental process involved in reproduction (GO: 0003006), reproduction (GO: 0000003), reproductive process (GO: 0022414), regulation of epithelial cell promotion (GO: 0050678), epithelial cell promotion (GO: 0050673), Embryonic genitalia morphogenesis (GO: 0030538) and luteolysis (GO: 0001554) confirmed that CP may have a significant impact on the reproductive function of offspring.

KEGG metabolic pathway results showed that the metabolic pathways related to inflammation were enriched, such as NF-*κ*B signaling pathway (OCU04064), inflammatory mediator regulation of TRP channels (OCU04750), toll like receiver signaling pathway (OCU04620), TNF signaling pathway (OCU04668), TGF beta signaling pathway (OCU04350), IL-17 signaling pathway (OCU04657) et al. As a major inflammatory regulator, NF-*κ*B signaling pathway can not only control the transcription of a series of inflammatory response related genes, but also regulate tight junction proteins closely related to permeability [61–64]. What is more, western blot results also confirmed the high expression levels of NOX4 and NF-*κ*B protein in CP group. Wang et al. showed that ovariectomy triggered NOX4 overexpression, followed by ROS overproduction and NF-*κ*B activation as well as mitochondrial dysfunction, which is demonstrated to function through the activation of NOX4/ROS/NF-*κ*B pathway [65]. CP lead to the increase of the relative abundance of some pathogenic microorganisms in the intestine, and further resulted in the enrichment of inflammatory factors and virulence factors in the host [66]. It may be brought about damage to the endometrial epithelial cells by activating the NOX4-ROS-NF-*κ*B pathway.

In addition, metabolic pathways related to reproduction and development, such as tight junction (OCU04530), estrogen signaling pathway (OCU04915), and Varian steroidogenesis (OCU04913), in which tight junction is the most characteristic structure of epithelial cells, and it is also an important structure for epithelial cell barrier function, maintaining cell polarity, and regulating the permeability of small molecules near cells, which is composed of a variety of membrane penetrating proteins [67, 68]. Relative to the Con group rabbits, CP treatment destroyed the tight junction structure of endometrial epithelial cells, and the relative expression level of tight junction factors ZO-1, OCLN, and CLDN1 was also significantly reduced (Figure 5). The expression levels of VCAM-1 and ICAM-1 in endometrial epithelial cells of CP group were significantly increased (Figures 5(a), 5(b), and 5(c)). Dehbi et al. showed that stimulation of proinflammatory factors can lead to increased expression of ICAM-1 and VCAM-1 on the plasma membrane, and facilitate with VLA4 to mediate the rapid transfer of leukocytes to inflammatory sites and participate in inflammation and its downstream reactions [69]. These results confirmed that the permeability of endometrial epithelial cells was increased, the tight junction structure and function were abnormal, and the endometrial barrier was damaged under CP. The expression of these tight junction factors in endometrial epithelial cells is very important for the maintenance of embryo implantation and developmental function [70, 71]. The reduction of tight junction factors in endometrial epithelial cells may lead to the dysfunction of uterine barrier, resulting in the increase of macromolecular solutes passing through the uterine barrier, aggravating placental function damage or leading to adverse outcomes in newborn rabbit [72, 73].

## 5. Conclusions

Coprophagy is an instinctive behavior for rabbits to maintain their growth and reproductive performance. CP can affect the level of inflammation and oxidative stress, activate the NOX4-ROS-NF-*κ*B pathway of endometrial epithelial cells of pregnant New Zealand white rabbits, cause the disintegration of tight junction proteins in endometrial epithelial cells of New Zealand white rabbits, increased the expression levels of endometrial adhesion proteins ICAM-1 and VCAM-1 and lead to the damage of uterine tissue morphology, and finally lead to the reduction of embryo implantation rate and the adverse outcome of newborn rabbits.

## Figures and Tables

**Table 1:** Effects of coprophagy prevention on rabbit growth performance.

Items	Control group (*n* = 6)	CP group (*n* = 6)
Initial body weight (kg)	3.13 ± 0.10	3.00 ± 0.20
Average daily feed intake (g)	241.16 ± 16.32	227.13 ± 19.72
Average daily body weight gain (g)	23.60 ± 3.30^A^	15.95 ± 3.47^B^
Final weight (kg)	5.12 ± 0.26^A^	4.34 ± 0.29^B^
Ratio of feed intake/body weight (%)	10.38 ± 1.66^A^	14.88 ± 1.02^B^

Note: data in the same row are marked with different capitals A and B, indicating significant difference (*P* value < 0.05).

**Table 2:** Effects of coprophagy prevention on rabbit slaughter performance.

Items	Control group (*n* = 6)	CP group (*n* = 6)
All eviscerated weight (kg)	2.35 ± 0.04^A^	1.98 ± 0.18^B^
Half eviscerated weight (kg)	2.66 ± 0.09^a^	2.30 ± 0.20^b^
Oophoron weight (g)	1.12 ± 0.11	1.15 ± 0.24
Uterus weight (g)	90.60 ± 15.98^A^	78.77 ± 11.89^B^
Placenta weight (g)	103.25 ± 10.69	82.33 ± 21.52

Note: data in the same row are marked with different capitals A and B, indicating significant difference (*P* value < 0.05).

**Table 3:** Effects of coprophagy prevention on rabbit reproduction performance.

Items	Control group (*n* = 6)	CP group (*n* = 6)
Breeding rate (%)	75.00	40.91
Total number born	13.80 ± 2.77	13.17 ± 5.91
Number born alive	13.60 ± 2.41	12.00 ± 5.62
Live births rate (%)	100.00 ± 0.00^A^	88.23 ± 4.14^B^
Stillbirth rate (%)	0.00 ± 0.00^A^	11.77 ± 4.14^B^
Birth litter weight (g)	652.40 ± 60.68^A^	508.33 ± 109.36^B^

Note: data in the same row are marked with different capitals A and B, indicating significant difference (*P* value < 0.05).

## Data Availability

The raw sequence files were deposited to the National Center for Biotechnology Information Sequence Read Archive with accession number PRJNA885444.

## Supplementary Materials

Supplementary Materials Supplementary Figure S1: quality and accuracy analysis of transcriptome sequencing data. (A) Expression patterns of all genes in the sample. (B) Correlation of gene expression levels between samples. (C) Principal component analysis results of all samples. Supplementary Figure S2: verification of the accuracy of transcriptome sequencing results. (A) RT-qPCR results. (B) Differential multiples of gene expression in transcriptome sequencing. Supplementary Table S1: the diet composition of rabbits. Supplementary Table S2: the primers for RT-qPCR. Supplementary Table S3: RT-qPCR system and procedure. Supplementary Table S4: transcriptome sequencing data output report. Supplementary Table S5: analysis of differentially expressed genes. Supplementary Table S6: enrichment analysis of GO Terms. Supplementary Table S7: enrichment analysis of KEGG metabolic pathway.
