# Supplementary material for: Coprophagy Prevention Affects the Reproductive Performance in New Zealand White Rabbits Is Mediated through Nox4-ROS-NFκB Pathway
Source: Oxid Med Cell Longev. 2022 Dec 21;2022:8999899. doi: 10.1155/2022/8999899 (PMC11401658; doi:10.1155/2022/8999899)
Supplement: Supplementary Materials — Supplementary Figure S1: quality and accuracy analysis of transcriptome sequencing data. (A) Expression patterns of all genes in the sample. (B) Correlation of gene expression levels between samples. (C) Principal component analysis results of all samples. Supplementary Figure S2: verification of the accuracy of transcriptome sequencing results. (A) RT-qPCR results. (B) Differential multiples of gene expression in transcriptome sequencing. Supplementary Table S1: the diet composition of rabbits. Supplementary Table S2: the primers for RT-qPCR. Supplementary Table S3: RT-qPCR system and procedure. Supplementary Table S4: transcriptome sequencing data output report. Supplementary Table S5: analysis of differentially expressed genes. Supplementary Table S6: enrichment analysis of GO Terms. Supplementary Table S7: enrichment analysis of KEGG metabolic pathway. [file 8999899.f1.zip › Supplementary Table S4.docx]

Supplementary Table S3 Transcriptome sequencing data output report

| Samples | Bases (bp) | Q30 (%) | Clean_data (bp) | Total_Mapped |
| --- | --- | --- | --- | --- |
| CON 1 | 8163282000 | 92.81 | 7620871200 | 45629420 (89.81%) |
| CON 2 | 7399391700 | 92.98 | 6904784100 | 41328214 (89.78%) |
| CON 3 | 8421834900 | 93.03 | 7847747100 | 46877879 (89.60%) |
| CP 1 | 8144972700 | 92.53 | 7607071800 | 45631708 (89.98%) |
| CP 2 | 7367317200 | 92.50 | 6860209200 | 41517395 (90.78%) |
| CP 3 | 7465977300 | 92.21 | 6956928900 | 41627236 (89.75%) |
